# The FP25K Acts as a Negative Factor for the Infectivity of AcMNPV Budded Virus

**DOI:** 10.1371/journal.pone.0128471

**Published:** 2015-05-28

**Authors:** Shufen Li, Manli Wang, Shu Shen, Zhihong Hu, Hualin Wang, Fei Deng

**Affiliations:** State Key Laboratory of Virology, Wuhan Institute of Virology, Chinese Academy of Sciences, Wuhan, P.R. China; Wuhan University, CHINA

## Abstract

Baculoviruses generally produce two progeny phenotypes—the budded virus (BV) and the occlusion-derived virus (ODV)—and the intricate mechanisms that regulate the temporal synthesis of the two phenotypes are critical for the virus replication cycle, which are far from being clearly understood. FP25K was reported to be responsible for the regulation of BV/ODV, and the mutations within result in a decrease of normal ODVs formation and an increase of BVs production. In this study, we demonstrated that the increase of BV titer in an *fp25k* knockout recombinant (*fp25k*-negative) was a result of higher infectivity of BVs rather than an increased production of BVs. The constitution of the major structural proteins and genome of parental and *fp25k*-negative BVs were analyzed. The results showed that the integrity of the majority of DNA packaged into the *fp25k*-negative BVs was intact; i.e., the genomic DNA of *fp25k*-negative BV had better transformation and transfection efficiency than that of the parental virus, indicating more intact genomes in the virions. Although the analysis of proteins associated with BVs revealed that more envelope protein GP64 were incorporated into the *fp25k*-negative BVs, subsequent experiments suggested that overexpression of GP64 did not improve the titer of BVs. Thus, we conclude that the main reason for higher infectivity of BVs is due to better genome integrity, which benefits from the deletion of *fp25k* resulting in increased stability of the genome and produce a higher proportion of infectious BVs. FP25K acts as a negative factor for the infectivity of BV.

## Introduction

Baculoviruses are a diverse group of large double strain DNA viruses targeting insects, which contain four genera: *Alphabaculovirus*, *Betabaculovirus*, *Gammabaculovirus*, and *Deltabaculovirus*. Except for members of the genus *Gammabaculovirus*, two morphologically distinct virion phenotypes are produced in the biphasic life cycle of baculoviruses: the budded virus (BV) and the occlusion-derived virus (ODV) [1, 2]. ODV enters the epithelial cells of the insect midgut through direct membrane fusion and initiates primary infection, while BV is transmitted from cell to cell, and is responsible for secondary and systemic infection [3, 4]. Baculoviruses have been successfully developed as bioinsecticides or eukaryotic expression vectors/gene therapy vectors. Genetic modification was applied to improve baculovirus as a more efficient expression vector [5].

During the life cycle of baculoviruses, progeny nucleocapsids of the Autographa californica multiple nucleopolyhedrovirus (AcMNPV) begin to egress from the nucleus after assembly in the intra nuclear virogenic stroma at about 18 h post-infection [6]. Then nucleocapsids bud through the plasma membrane, obtaining a lipid-containing envelope derived from the membrane with glycoproteins, and finally become progeny BVs [7]. In the very late phase of the life cycle, nucleocapsids are retained in the ring zone of the nucleus to become enveloped by intra nuclear microvesicles to form ODVs, and finally to be occluded in a crystalline matrix called the polyhedra [3]. To date, the ODV components of AcMNPV [8] and three other baculoviruses, Helicoverpa armigera single nucleocapsid polyhedrovirus (HearNPV) [9], Culex nigripalpus nucleopolyhedrovirus (CuniNPV) [10], and Bombyx mory ucleopolyhedrovirus (BmNPV) [11], have been analyzed by mass spectrometry-based techniques. A comprehensive proteomics analysis of AcMNPV BV-associated proteins was reported [12]. Comparative proteomics have been recently used to reveal differences in protein compositions between the two phenotypes of HearNPV [13].

Serial passage of nucleopolyhedrovirus (NPVs) in cultured cell lines could result in few polyhedra (FP) phenotype that was first observed in infected *Trichoplusiani* cells [14]. FP phenotypes usually lose part of the viral genome or acquire a few of host genome fragments through transposon site. Mutations within the *fp25k* gene were identified to be responsible for the FP phenomenon of AcMNPV [15]. Cells infected with FP mutants produced BVs with higher titer and smaller numbers of occlusion bodies [16]. Braunagel *et al*. observed that mutations within the *fp25k* gene resulted in a remarkable change in the accumulation of several baculovirus structural proteins, including GP64, ODV-E26 and ODV-E66 [17]. The expression level of ODV-E66 decreased in the cells infected with FP mutants, whereas production of GP64 and ODV-E26 increased. In addition, FP25K was shown to interact with ODV-E26, ODV-E66 and GP64, and form a complex with ODV-E25, ODV-E66 and VP39. FP25K and the protein complexes associated with it may participate in the intracellular transport of viral proteins and contribute to ODV formation [17]. Deletion of FP25K decreased the accumulation of E66 protein and blocked the transport of E66 to inner nuclear membrane [18]. Further investigations indicated that transport of ODV-E66 to the inner nuclear membrane is mediated via a sorting motif, facilitated by FP25K and other viral proteins [19].

Like FP phenomenon, the defective interfering particle (DIP) mutants, which are missing part of the genome and thus are replication-defective, accumulate in cell culture during virus passage [20]. It has been reported that transposon insertion could be a crucial step in DIP generation during serial passage [20, 21]. A recent report found that the production of baculovirus DIPs during serial passage could be delayed when the target sites for transposon insertion were deleted from the *fp25k* gene [22]. These results suggest a potential relation between *fp25k* mutant and genome stability.

Previous studies have indicated that the *fp25k* gene might be involved in the regulation of BV and ODV ratio and, ultimately, the yield of the two virion phenotypes [23]; however, the precise molecular mechanism behind this remains unclear. In this study, in order to investigate the specific role of FP25K in the formation of BV and ODV and to further improve the baculovirus as an expression vector, the *fp25k* gene was knocked out from the genome of vAcΔ*cc*, which was deficient in *chitinase* and *v-cathepsin* gene and proved to be a expression vector had positive influence on the integrity and production of intracellular or secreted proteins [24, 25]. We found that the deletion of *fp25k* gene caused a higher BV titer and a decreased ODV formation. Further investigation indicated that the increased BV titer was due to higher infectivity. The constitution analyses of the major structural proteins and viral genomes of both parental and *fp25k*-negative BVs suggested that more envelope proteins and higher proportion of genomes with intact integrity were incorporated into *fp25k-*negative BVs. Since overexpression of GP64 could not result in an improvement in the titer of BVs, we speculate that higher proportion of intact virus genomes incorporation is likely to be the main reason for the higher infectivity, and FP25K acts as a negative factor in this process.

## Materials and Methods

### Cell lines and viruses

The *Spodoptera frugiperda* (Sf9) cell line [26] (as gift from Prof. Just M. Vlak, Wageningen University, The Netherlands) was cultured in Grace’s insect medium (pH 6.0; Gibco-BRL), supplemented with 10% fetal bovine serum (FBS; Gibco-BRL) at 28°C. The AcBacΔ*cc* bacmid which was deficient in both *chitinase* and *cathepsin* genes [24] was generously provided by Prof. Just M. Vlak (Wageningen University, The Netherlands), and was propagated in *Escherichia coli* strain DH10β. Viruses were harvested from culture supernatants followed by purification (5,000×g for 5 min) to eliminate cell debris. Titers of recombinant AcMNPVs were determined by endpoint dilution assays (EPDA) with Sf9 cells [27].

### Construction of *fp25k*-knockout, repair and parental bacmids containing *egfp*

**Fig 1:**
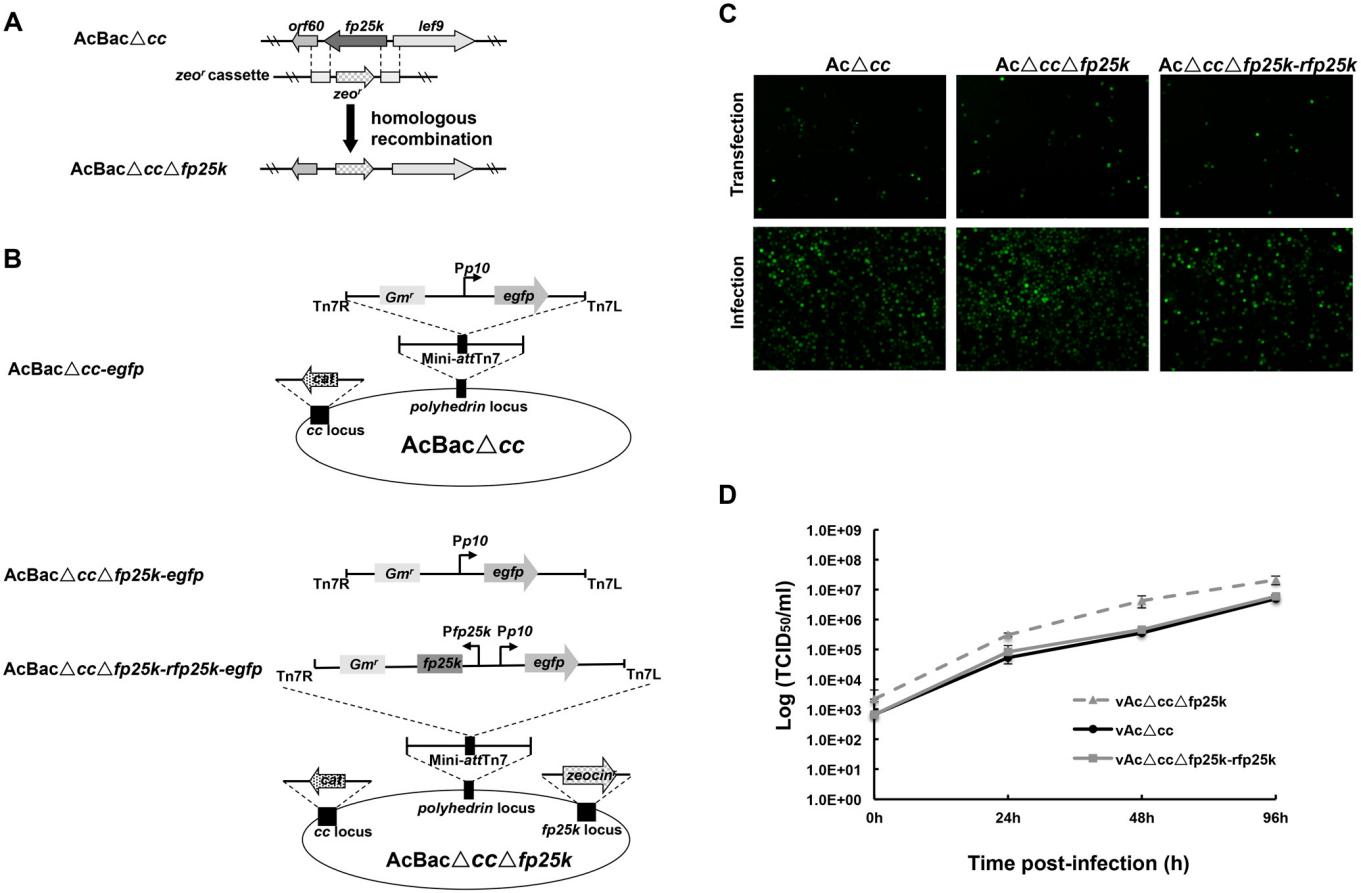
Characterization of recombinant bacmids and viruses. (A) Construction of *fp25k* knockout bacmid. The *fp25k* gene in AcBacΔ*cc* bacmid was deleted and replaced with the zeocin resistance gene (*zeocin* ^*r*^) through homologous recombination. (B) Strategy for construction of recombinant viruses, vAcΔ*cc*, vAcΔ*cc*Δ*fp25k* and vAcΔ*cc*Δ*fp25k-rfp25k*, the e*gfp* gene was inserted into the *polyhedrin* locus by transposition. (C) Transfection-infection assay of recombinant bacmids for viral propagation. At 48 h p.t., GFP-expressing cells were observed by fluorescence microscopy (upper panel). The fluorescent signals of GFP in cells infected by transfection supernatant were visualized at 72 h p.i. (lower panel). (D) One step growth curves of vAcΔ*cc*, vAcΔ*cc*Δ*fp25k* and vAcΔ*cc*Δ*fp25k-rfp25k*. Sf9 cells were infected with each virus at an MOI of 5, the supernatants were harvested at 0, 24, 48 and 96 h p.i. and determined for the production of infectious virus by EPDA. The results were transformed logarithmically. Each point represents the average titer from three independent infections. Error bars represent standard deviations.

The *fp25k* gene of AcBacΔcc bacmid was knocked out by homologous recombination in *E*. *coli* BW25113 containing AcBacΔcc bacmid, in accordance with the method of Hou *et al*. [28], replacing the *fp25k* gene by the zeocin-resistance gene (*zeo* ^*r*^). Briefly, a 444 bp sequence upstream of the *fp25k* gene was amplified by PCR with the forward primer (5’-AAGCTTTGTCTGTAAACTTGTTGGTCT-3’; *Hin*dIII site underlined) and the reverse primer (5’-GAATTCGGCGCCTTGAGCAGAGACACGTTAATC-3’; *Eco*RI and *Nar*I sites underlined). A 230 bp sequence downstream of the *fp25k* gene was obtained with the forward primer (5’-GGCGCCTGAGTCTGAAAACGATAGCG-3’; *Nar*I site underlined) and the reverse primer (5’-GCTAGCTTTTTCAAATATCCTCTTACCG-3’; *Nhe*I site underlined), using AcMNPV genome DNA as template. The PCR products were cloned into a pFastBac-Dual vector (Invitrogen, USA). The *zeo* ^*r*^ gene was further cloned into the pFastBac-Dual vector using the *Nar*I site, generating the transfer vector pFastBac-Dual-D*fp25k*. This transfer vector was digested by *Hin*dIII and *Nhe*I, and the linear fragment containing *zeo* ^*r*^ and the flanking sequences of the *fp25k* gene was used to transform BW25113 competent cells containing AcBacΔ*cc* bacmid with the helper plasmid pKD46. Positive clones were selected through both zeocin and kanamycin resistance. The construction strategy was illustrated in Fig 1A. The correct bacmid clone was verified by PCR using primers flanking the *fp25k* locus.

In order to observe the transfection and infection directly, an *egfp* gene under the control of the *p10* promoter was inserted through transposition into the polyhedrin gene locus of AcBacΔ*cc* and AcBacΔ*cc*Δ*fp25k* bacmids. A fragment of *fp25k* gene with its own promoter and *egfp* gene driven by the *p10* promoter was inserted into the polyhedrin gene locus of AcBacΔ*cc*Δ*fp25k* bacmid to generate the *fp25k* repair bacmid containing *egfp* (Fig 1B).

### Transfection and infection assays

Sf9 cells (2×10^6^) were cultured in 35 mm diameter tissue culture dishes, and transfected with each recombinant bacmid DNA (approximately 10 μg) using 10 μl of Lipofectin (Invitrogen, USA) according to the manufacturer's specification. At 48 h post transfection (p.t.), cells were examined for green fluorescent protein (GFP) expression by fluorescence microscopy. For the infection assay, at 5 days p.t., supernatants from the transfections were harvested and centrifuged at 5000 rpm for 5 min to remove cell debris, and then 200 μl of the supernatant were used to infect fresh Sf9 cells. Cells were monitored by fluorescence microscopy at 72 h post infection (p.i.).

### One-step virus growth curve

Sf9 cells (1×10^6^ per well; six-well plates) were infected with each recombinant virus at a multiplicity of infection (MOI) of 5. At corresponding time post infection, 15 μl of the supernatants from infected cells were collected, and the titers of each time points were determined by EPDA in Sf9 cells [27]. GFP was the marker of infection used during the assay. All infection experiments and EPDA were performed three times, and the growth curves were generated by the arithmetic mean data of three independent infections.

### Quantitative PCR analysis of genomic DNA copies in BVs and infected cells

At 0, 18, 24, 48, 72 and 96 h p.i., 50 μl of the infected cell culture supernatants were collected to isolate BV DNA as previously described [29]. For quantitative PCR (qPCR) analyses, 5 μl of BV DNA were used as a template to determine BV genomic DNA copies as previously described method [30].

For identification of total virus genomic DNA copies in infected cells, 1×10^6^ cells were infected with each recombinant virus (5 MOI) and total cellular DNA was isolated at 0, 72 and 96 h p.i. using a commercial kit (Genomic DNA Rapid Isolation Kit; BioDev, China). 5 μl isolated total cellular DNA was used as template in qPCR analyses to determine viral copy numbers in infected cells with primers of viral gene *vp80*: *vp80*-For: 5’-gacgatgtcgttaatcgtgc-3’ and *vp80*-Rev: 5’-atcagcatcgctattcagataa-3’. The measured virus genomic DNA copies in both recombinants infected cells of each time points were compared.

### Electron microscopy

Sf9 cells (2×10^6^) were infected with vAcΔ*cc* or vAcΔ*cc*Δ*fp25k* (5 MOI). Cells were harvested at 48, 64, 72 and 96 h p.i., and washed three times with phosphate-buffered saline (PBS). All samples were fixed with 2.5% (w/v) glutaraldehyde in 0.1 M sodium phosphate, and processed for transmission electron microscopy (TEM) as described previously [29]. ODV formation in infected cell was observed by TEM (FEI Tecnai G2 microscope; 200 kV).

### Western blot analysis

Sf9 cells (2×10^6^) infected with recombinant vAcΔ*cc*/ vAcΔ*cc*Δ*fp25k* at 5 MOI were collected at 48 h p.i. and rinsed with PBS. The protein samples were separated through 12% sodium dodecyl sulfate-polyacrylamide gel electrophoresis (SDS-PAGE) and transferred onto PVDF membranes (Millipore Corporation, USA) by semi-dry electrophoresis. The Western blot analyses were performed with primary polyclonal antibodies generated from rabbit which against AcMNPV structural proteins: BV envelope protein GP64 and Ac23 [30], nucleocapsid protein AC109 (generated in our lab, unpublished data) and 38K [31].

The structural proteins incorporated into BV were identified by Western blot analyses. Genomic DNA was isolated from 100 μl of BV supernatant for each recombinant virus, and quantified by qPCR as described above. BVs containing equal copies of genome were centrifuged at 13,000 rpm for 30 min at 4°C. Samples were disrupted under reducing condition (4×SDS-PAGE sample buffer, 100°C) and separated by SDS-PAGE (12% separation gel). The polyclonal antibodies against AcMNPV structural proteins: GP64, Ac23 and VP39 [12] were used as primary antibodies for Western blot analyses. The experiment was performed as described previously [30].

### Quantitative reverse transcription PCR analysis

Sf9 cells (2×10^6^) in 35-mm diameter tissue culture dishes were infected with vAcΔ*cc* or vAcΔ*cc*Δ*fp25k* (5 MOI). At 48 h p.i., total RNA was isolated with TRIzol (Invitrogen, USA) and subsequently treated with RQ1 RNase-Free DNase (Promega, USA) to digest the residual DNA. A two-step quantitative reverse transcription (qRT)-PCR method was performed using 0.5 μg of DNA-free RNA as template. The first step of the cDNA synthesis was performed using M-MLV Reverse Transcriptase (Promega, USA) and oligo (dT) primers (5’- CTGATCTAGAGGTACCGGATCCTTTTTTTTTTTTTTT-3’). The second step of cDNA qPCR using 1 μl template cDNA was performed as previously described [30]. The qRT-PCR primer pairs were derived from the *gp64*, *38k*, *Ac109* and *polyhedrin* genes. 28S rRNA was used as an internal control (Table 1). Relative viral gene RNA levels were calculated as the quantity of the specific gene RNA normalized to 28S rRNA levels. Each experiment was performed three times.

**Table 1:** Primers used for quantitative reverse transcription PCR.

Primer	Sequence
*28s*for	5’GGTTGCTTGAGAGTGCAGCC3’
*28s*rev	5’TTCATTCGAGTTTCGCAGGT3’
*38k*for	5’CGCACGTGGTCGTGTTTGA3’
*38k*rev	5’GTCTCGCATCGAGTGTGCT3’
*gp64*for	5’CAGGCGTATGCGTACAACGG3’
*gp64*rev	5’ACAGTCGTCGCTGTCACTGC3’
*ac109*for	5’ATGGAGTGCCCGTTTCAGATT3’
*ac109*rev	5’TTGGCGATCGACTGTCTATGT3’
*polyhedrin*for	5’GTACCTACGTGTACGACAACAA3’
*polyhedrin*rev	5’GATTTCCTTGAAGAGAGTGAGTT3’

### BV Genomic DNA transformation and transfection assay

BV genomic DNA isolated from 100 μl of BVs harvested at 36 and 48 h p.i. was quantified by qPCR. Equal copies (5×10^8^) of *fp25k*-negative and control BV genomic DNA were used to transform competent *E*. *coli* DH10β cells by electroporation, and the number of colony forming units (CFUs) formed under kanamycin selection was calculated. The experiment was carried out for three times.

Genomic DNA isolated from 2 ml of BVs was dissolved in 50 μl of ddH_2_O, and then quantified by qPCR. 1×10^10^ copies of *fp25k*-deleted and control BV genome DNA were transfected into Sf9 cells. At 36 h p.t., cells were photographed under fluorescence microscopy. For each analysis, five fields were chosen randomly, and the number of cells expressing EGFP was calculated. The experiment was performed three times.

### Overexpression of GP64

For generation of vAcΔ*cc*-*gp64*, the extra *gp64* gene driven by *gp64* promoter and an *egfp* gene under the control of the *p10* promoter were inserted through transposition into the polyhedrin gene locus of AcBacΔ*cc*. The bacmid of AcBacΔ*cc-gp64* with an *egfp* gene was transfected into Sf9 cells as described above, and supernatant from the transfection was harvested to infect a new batch of Sf9 cells to generate vAcΔ*cc*-*gp64*. The *gp64* expression in infected cells and GP64 incorporation in budded virions were detected as described above. One-step virus growth curves of vAcΔ*cc* and vAcΔ*cc*-*gp64* were conducted as described previously.

## Results

### Generation of recombinant viruses

The *fp25k* gene of AcBacΔ*cc* bacmid was successfully deleted and the recombinant named AcBacΔ*cc*Δ*fp25k* was verified by PCR detection (data not shown). An *egfp* gene under the control of the *p10* promoter was inserted into AcBacΔ*cc* or AcBacΔ*cc*Δ*fp25k* (Fig 1B). A repair bacmid, AcBacΔ*cc*Δ*fp25k-rfp25k*, was also generated by inserting *fp25k* with its own promoter and e*gfp* into the *polyhedrin* locus of AcBacΔ*cc*Δ*fp25k* to confirm the phenotype was resulting from the deletion of *fp25k* (Fig 1B). Sf9 cells transfected with either bacmid could express GFP at 48 h p.t. (Fig 1C). Infectious BVs were produced from each bacmid (Fig 1C). Expression of FP25K protein was undetectable in the cells infected with knockout virus vAcΔ*cc*Δ*fp25k* (data not shown).

In order to confirm the deletion of *fp25k* result in an increased yield of budded virus [16], Sf9 cells were infected with vAcΔ*cc*, vAcΔ*cc*Δ*fp25k* and vAcΔ*cc*Δ*fp25k*-*rfp25k*. The one-step growth curve of each virus is shown in Fig 1D. At 24 h p.i., the *fp25k*-negative virus had a higher BV titer than parental virus (vAcΔ*cc*), although the difference was not significant (*P*>0.05, analyzed by a two-tailed Student’s *t-*test). At 48 h p.i., the production of vAcΔ*cc*Δ*fp25k* is significantly higher than vAcΔ*cc* (*P<*0.05, analyzed by a two-tailed Student’s *t-*test). The *fp25k* repair virus showed a similar kinetic with the parental virus (Fig 1D). These results showed that the increased titer of *fp25k*-negative virus was due to the deletion of *fp25k*.

### The *fp25k*-negative virus produced more infectious progeny BVs

**Fig 2:**
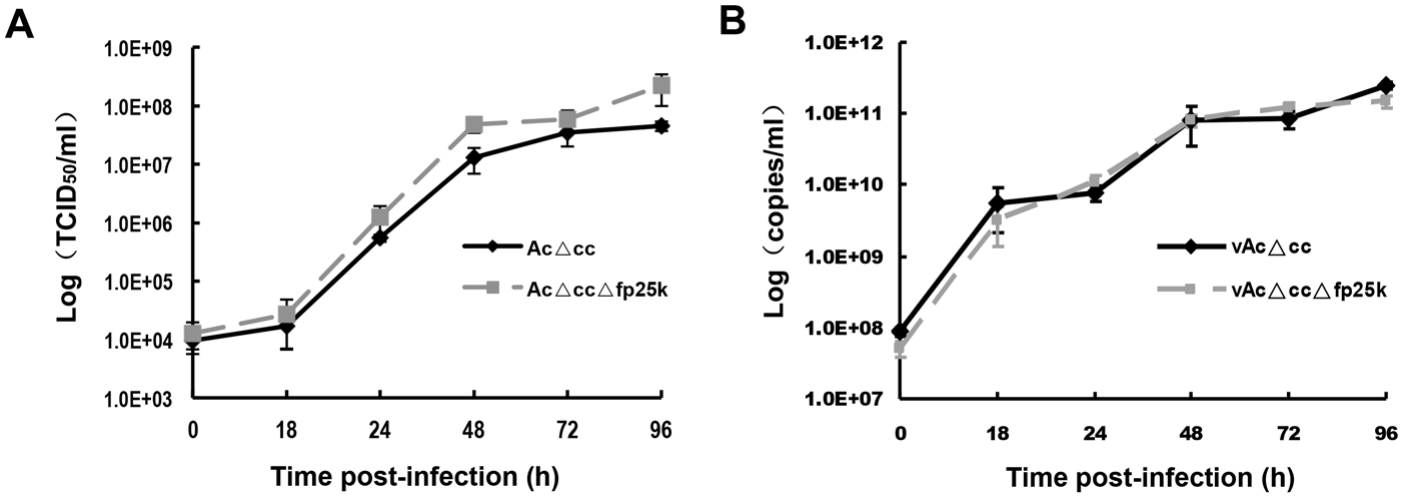
Virus titer determination and qPCR analysis of BV genomic DNA copy number in the supernatant. Sf9 cells were infected with vAcΔ*cc* or vAcΔ*cc*Δ*fp25k* at an MOI of 5. The supernatants from infected cells were collected at 0, 18, 24, 48, 72 and 96 h. p.i. Virus titers were determined by EPDA (A), genomic DNA was detected by qPCR (B), and the results were transformed logarithmically. Each point represents the average titer from three independent infections. Error bars represent standard deviations.

Both previous studies and our result indicated that *fp25k* mutant virus generated more BV during infection [16, 32], especially around the time point 48h p.i. (Fig 1D). In order to further investigate whether the deletion of *fp25k* facilitate virus production or infectivity, the virus genomic DNA copies were determined by qPCR in the same samples for One-step growth curve analysis (Fig 2A), we found that the similar genomic DNA copies were detected in both recombinants at each time points (Fig 2B). The result indicated that the titer of vAcΔ*cc*Δ*fp25k* was higher than vAcΔ*cc* whereas the copy number of genomic DNA was the same for each virus (Fig 2). At 48 h p.i., the average BV titer of vAcΔ*cc*Δ*fp25k* was about 3 times to that of vAcΔ*cc*. And at the same time point (48 h p.i.), the genomic DNA copies of the BVs in the supernatant were 8.1×10^10^ copies/ml for vAcΔ*cc* and similarly for vAcΔ*cc*Δ*fp25k* (8.0×10^10^copies/ml). Thus, the viral infectivity unit (copyies/TCID_50_) of vAcΔ*cc*Δ*fp25k* was calculated as 4.37×10^3^, and for parental virus vAcΔ*cc*, it was 1.14×10^4^. The result implied that *fp25k* gene deletion would lead to producing more infectious progeny BV particles.

### Non-enveloped nucleocapsids retained in the nucleus

**Fig 3:**
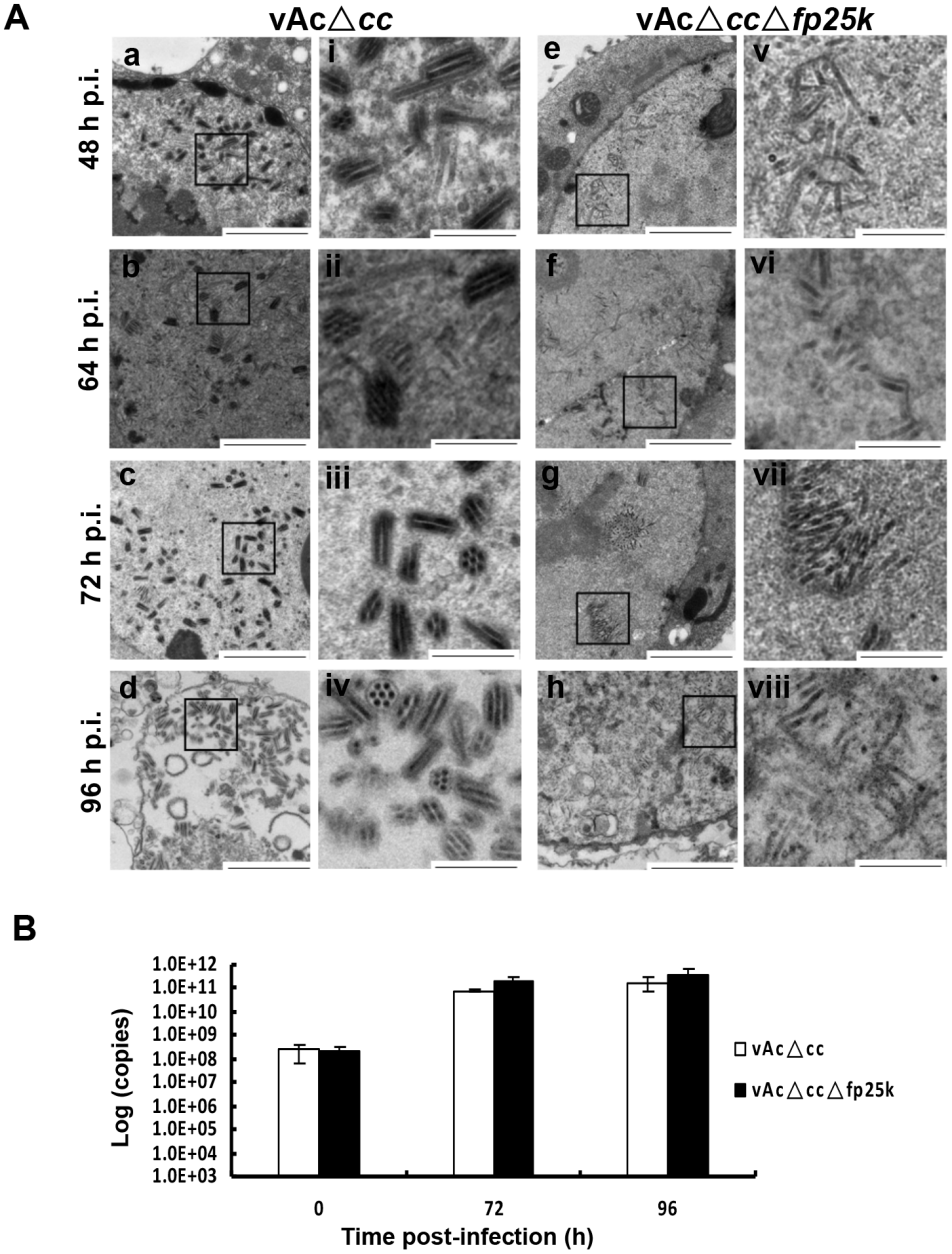
Cellular viral particles observation and determination. (A) Electron microscopy analysis. Sf9 cells were infected with vAcΔ*cc* or vAcΔ*cc*Δ*fp25k* at an MOI of 5. Infected cells were fixed with glutaraldehyde at 48, 64, 72 and 96 h p.i., and nucleocapsids occlusion was observed (i-viii). Enlargements of the blocked area in (a-h). Bars represent 1 μm and 250 nm. (B) Cellular viral particle determination. Sf9 cells were infected with vAcΔ*cc* or vAcΔ*cc*Δ*fp25k* (MOI = 5), total cellular DNA were collected at 0, 72 and 96 h p.i. and subjected to qPCR to determine the viral genome copy number. The data are from three independent experiments. Error bars represent standard deviation. Data were analyzed by two-tailed Student's *t*-test.

It has been reported that the envelopment of nucleocapsids within the nucleus of cells infected with FP mutant was incomplete [16]. We observed that the nucleocapsids envelopment and ODV formation of *fp25k*-negative virus also appeared to be significantly altered compared with control virus. Electron microscopy revealed a large number of completely enveloped nucleocapsids at the ring zone of Sf9 cells infected with control virus (48, 64, 72 and 96 h p.i.), while in cells infected with *fp25k*-negative virus, envelopment of nucleocapsids was impeded significantly (Fig 3A). It is important to note that nucleocapsids were not enveloped within ODVs did not participate in BV formation either, as they were still retained in the nucleus at corresponding time points during infection.

In addition, qPCR was carried out to determine the number of viral particles maintained in infected cells at 72 and 96 h p.i. We found that the number of viral genome copies in cells infected with *fp25k*-negative and control virus showed no significant difference (*P*>0.05, analyzed by a two-tailed Student’s *t-*test) (Fig 3B). The results of the assessment of BV titer, BV genome copy number in supernatant and infected cells as well as the EM observation indicated that vAcΔ*cc*Δ*fp25k* was more infectious than vAcΔ*cc*, rather than having a higher absolute production compared to the control.

### The expression of *gp64*, *ac109* and *polyhedrin* genes were regulated by FP25K at transcriptional level

**Fig 4:**
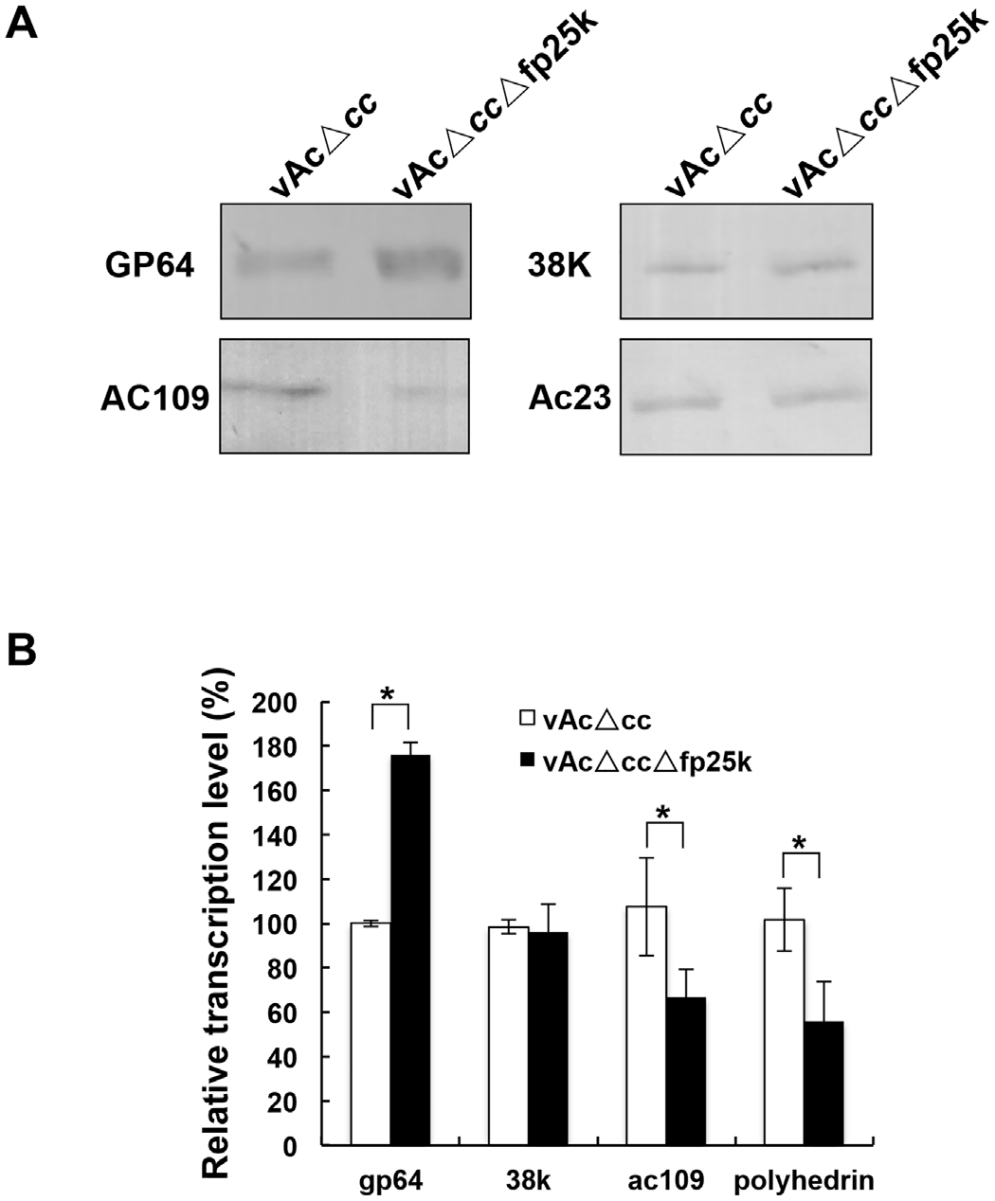
Expression analyses of viral structural proteins in infected cells. (A) Sf9 cells were infected with vAcΔ*cc* or vAcΔ*cc*Δ*fp25k* at an MOI of 5. Infected cells were collected at 48 h p.i. and analyzed by western blotting using the corresponding antibodies, the results were repeated twice. (B) Sf9 cells infected with vAcΔ*cc* or vAcΔ*cc*Δ*fp25k* (MOI = 5) were collected at 48 h p.i. and analyzed by qRT-PCR. The transcriptional levels of viral genes were normalized to the internal control *28S* rRNA, and the transcription difference between vAcΔ*cc*- and vAcΔ*cc*Δ*fp25k*-infected cells was analyzed by the 2^*-ΔΔC*^T method. The results of corresponding genes in vAcΔ*cc*-infected cells were set as 100%. The data are from three independent experiments. Error bars represent standard deviation. Data were analyzed by two-tailed Student's *t*-test. * *P*<0.05.

It has been reported that mutation within AcMNPV *fp25k* increased the accumulation of GP64 and decreased production of ODV-E66 [17]. Sf9 cells infected with vAcΔ*cc*/vAcΔ*cc*Δ*fp25k* were harvested at 48 h p.i. to investigate whether the synthesis of other proteins was affected by *fp25k* deletion. Western blots were performed to detect the accumulation of structural proteins GP64, AC109, Ac23 and 38K. Expression levels of AC109 decreased significantly in cells infected with *fp25k*-negative virus, whereas synthesis of GP64 increased. The deletion had no effect on the expression of *ac23* and *38k* (Fig 4A). VP39 was used as an internal control to normalize the expression level (data not show).

Furthermore, qRT-PCR analysis was performed. Total RNA in cells infected with vAcΔ*cc* or vAcΔ*cc*Δ*fp25k* was isolated and reverse transcribed into cDNA for qPCR detection. The result showed that the transcription levels of *ac109* and *polyhedrin* genes were down regulated significantly (*P*< 0.05, analyzed by a two-tailed Student’s *t-*test), while the *gp64* gene was up regulated (*P*< 0.05, analyzed by a two-tailed Student’s *t-*test) (Fig 4B). It suggested that the expression of *gp64*, *ac109* and *polyhedrin* were regulated by FP25K at transcriptional level.

### Higher level of GP64 was incorporated into *fp25k*-negative BV particles

**Fig 5:**
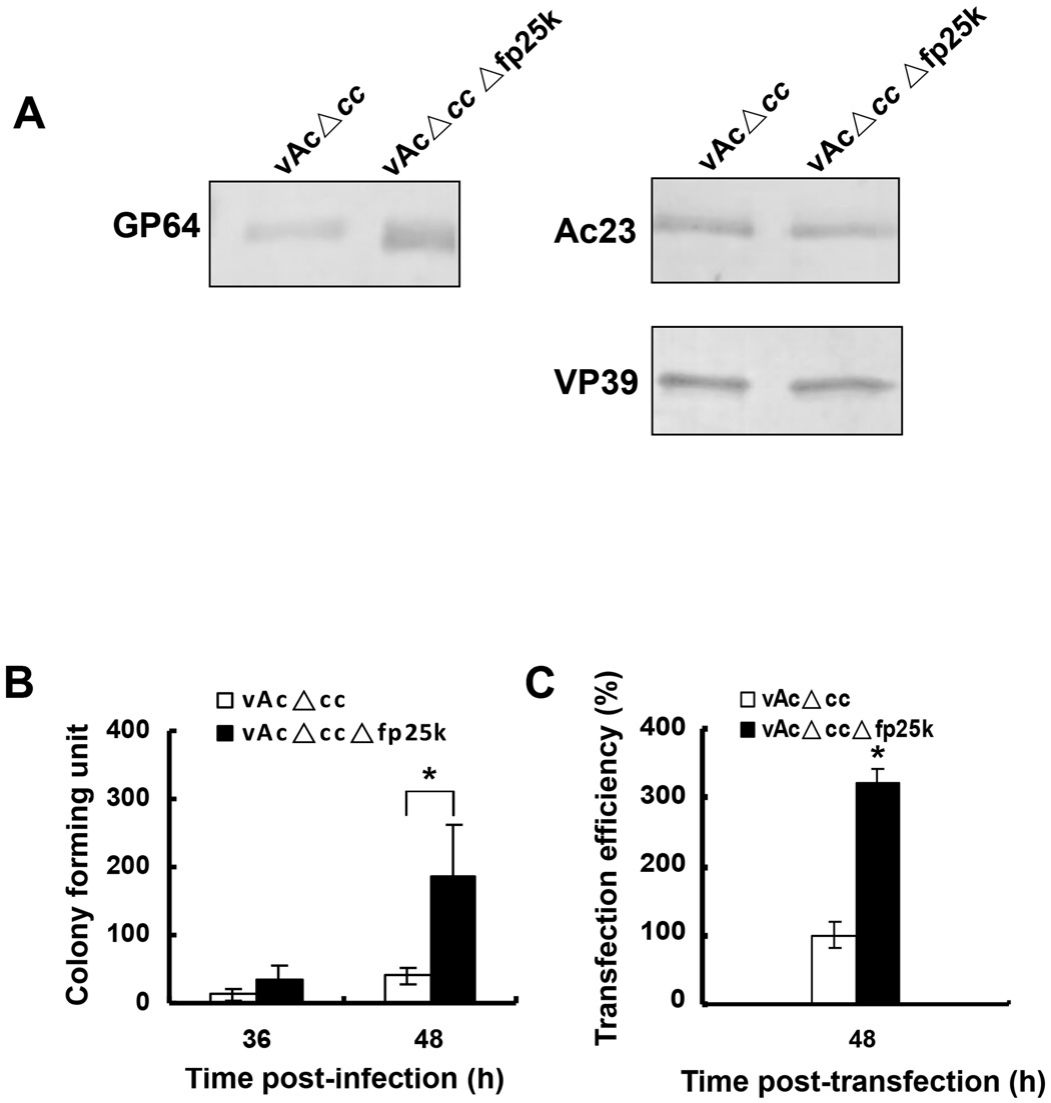
BV structural proteins incorporation and genome integrity analysis. (A) Western blot analysis of structural proteins incorporated into recombinant BVs. Equal copy numbers of vAcΔ*cc* and vAcΔ*cc*Δ*fp25k* virions were purified from the supernatants of infected Sf9 cells at 48 h p.i. and subjected to SDS-PAGE, then blotted with antibodies against GP64, Ac23, and VP39. (B) Transformation assay. Genomic DNA isolated from vAcΔ*cc* or vAcΔ*cc*Δ*fp25k* BVs harvested at 36 h and 48 h p.i. was quantified by qPCR. Equal copy numbers (5×10^*8*^) of genomic DNA was used to transform competent *E*. *coli* DH10B cells, and the number of CFUs was calculated. (C) Transfection assay. Equal copy numbers of genomic DNA isolated from vAcΔ*cc* and vAcΔ*cc*Δ*fp25k* BVs were transfected into Sf9 cell. Transfection efficiency was calculated as the number of cells expressing EGFP. The mean number of cells transfected by BV genomic DNA of control virus was set as 100%. Data are representative of three independent experiments. Error bars represent standard deviation. Data were analyzed by two-tailed Student’s *t*-test. * *P*<0.05.

Since the infectivity of *fp25k*-negative BV was higher than that of the control virus, we questioned whether the deletion confers alterations in the BV structure resulting in higher infectivity. Western blot analysis showed that the synthesis of BV envelope proteins GP64 increased in the *fp25k*-negative virus, we decided to investigate if higher amounts of the protein become incorporated into the BVs. Real-time qPCR were performed to determine the level of virus particles in supernatants. At 48 h p.i., supernatants containing equal copies (5×10^10^) of vAcΔ*cc* and vAcΔ*cc*Δ*fp25k* were collected and used in western blot analyses. As shown in Fig 5A, significantly higher levels of GP64 was detected in the vAcΔ*cc*Δ*fp25k* BVs compared with the vAcΔ*cc* BVs, while the incorporations of envelope protein Ac23 and nucleocapsid protein VP39 were unaltered.

### The overall genomic integrity of the *fp25k*-negative BVs was better than that of parental virus

The genomic stability of AcMNPV was shown to increase when the transposon insertion sites in *fp25k* gene was modified [22]. Since in our study the entire *fp25k* gene including the transposon insertion site was knocked out, we desired to find out whether the higher infectivity of *fp25k*-negative virus was due, at least part to better genomic stability. Genomic DNA was isolated from 100 μl of BV supernatant of *fp25k*-negative virus and control virus and quantified by qPCR. Genomic DNA of each virus in an equal copy number (5×10^8^) was transformed into competent *E*. *coli* DH10β cells, and the number of CFUs was calculated (Fig 5B). Genomic DNA formed colonies under antibiotic selection was considered to be complete circular DNA according to the fundamental principle of molecular cloning, since only complete circular DNA containing an antibiotic resistance gene can replicate in *E*. *coli* cells and confer on bacteria the ability to survive and proliferate in the selective growth medium with corresponding antibiotic [33]. The result indicated that more number of CFUs was formed when *E*. *coli* DH10βwas transformed with the *fp25k*-negative genomic DNA (*P*<0.05, analyzed by two-tailed Student’s *t*-test), suggesting more intact virus genomes were incorporated into the *fp25k*-negative virus particles.

The result of genomic integrity was further confirmed by transfection assay using the host cell line. Equal copy numbers (1×10^10^ copies) of *fp25k*-negative and control BV genomic DNA were used to transfect Sf9 cells. The transfection efficiency was calculated as the number of cells expressing GFP at 36 h p.t. The mean number of cells transfected by BV genomic DNA of control virus was set as 100% (Fig 5C). The transfection efficiency of *fp25k*-deleted genomic DNA was significantly higher than that of control DNA (*P*<0.05, analyzed by two-tailed Student’s *t*-test). Therefore, both results of transformation and transfection assays indicated that a higher proportion of intact viral genome was incorporated into *fp25k*-negative virus.

### Overexpression of GP64 could not enhance the BV titer

**Fig 6:**
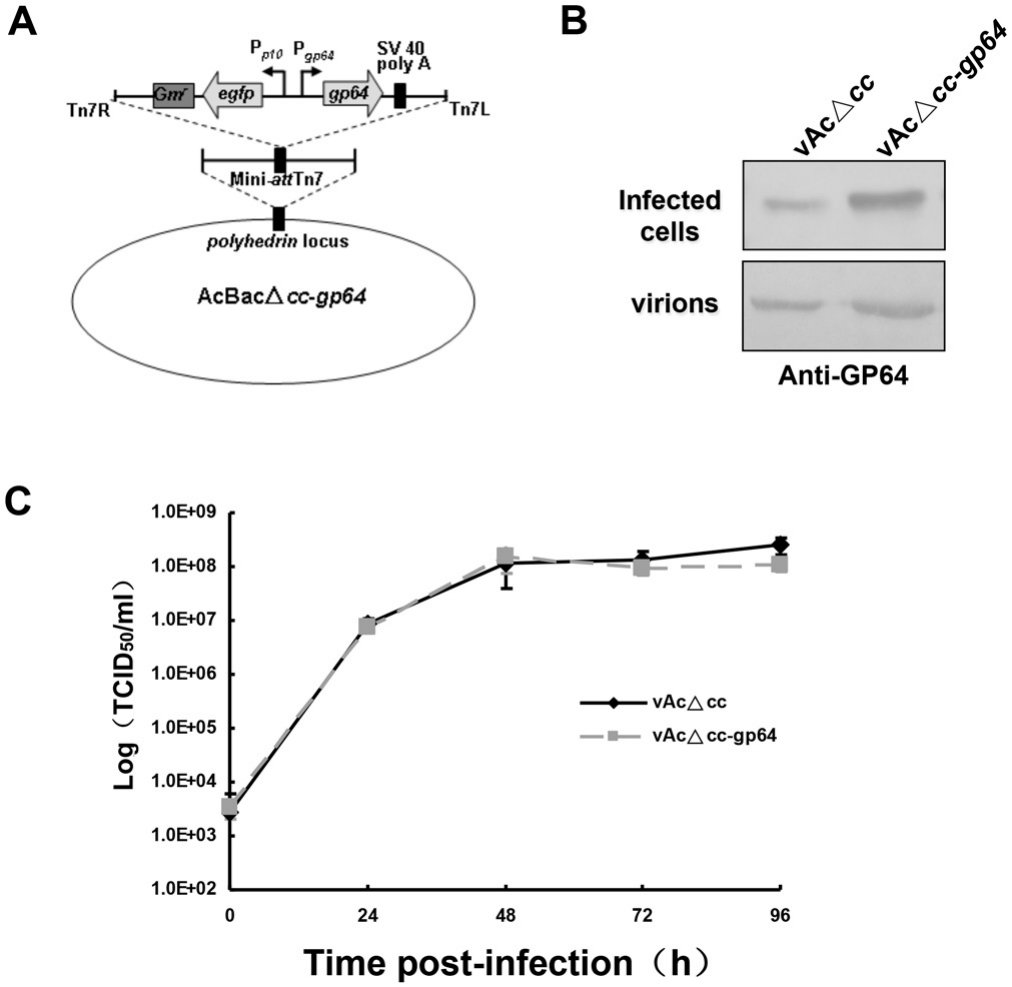
Overexpression of GP64. (A) Genomic organization of AcBacΔ*cc*-*gp64*. (B) Western blot analysis of cells infected with vAcΔ*cc* or vAcΔ*cc*-*gp64* and virions of vAcΔ*cc* or vAcΔ*cc*-*gp64*. For expression of GP64 in infected cells, Sf9 cells were infected with vAcΔ*cc* or vAcΔ*cc*-*gp64* at an MOI of 5. Infected cells were collected at 48 h p.i. and analyzed by western blotting. For GP64 incorporation into virions, equal copy numbers of vAcΔ*cc* and vAcΔ*cc*-*gp64* virions were purified from the supernatants of infected Sf9 cells at 48 h p.i. and subjected to SDS-PAGE, then blotted with antibody against GP64, experiments were repeated twice. (C) One-step growth curve of vAcΔ*cc* and vAcΔ*cc*-*gp64*, virus titers were determined by EPDA. Data are representative of three independent experiments. Error bars represent standard deviation.

To investigate whether the increased infectivity of *fp25k*-negative virus benefited from a higher level of GP64 incorporation, GP64 was overexpressed under its native promoter in vAcΔ*cc*-*egfp*. Western blot analysis of infected cells verified that GP64 was successfully overexpressed (Fig 6B), and that overexpressed GP64 was incorporated into BVs (Fig 6B). However, the results of the one-step growth curve assay showed that the recombinant virus titer was not increased by overexpression of GP64 (Fig 6C), suggesting that a higher level of GP64 incorporation to BVs might not be the major reason for the enhancement of infectivity of BV.

## Discussion

FP mutants of baculoviruses often result from acquisition of host cell DNA fragments or loss of a portion of the viral genome [34]. The common characteristics of the FP phenomenon are a decrease in the number of OBs, an increase in the production of BVs, and reduced numbers of completely enveloped ODVs [16, 35]. In our investigations, an *fp25k*-negative mutant virus was constructed. In comparison with the control virus, the mutant produced BVs with higher infectious titer than the parental virus, corroborating previous data [16, 32]. There are at least two hypotheses to explain this increased BV production: (1) that the nucleocapsids destined to form ODVs actually participate in BV formation, or (2) *fp25k*-negative virus produced BVs with higher infectivity [16]. We found that the number of virus genome copies in supernatant did not increase, when deletion of *fp25k* caused an increase in BV titer (Fig 2). EM observation of infected cells at different time points revealed that only few normal ODV formed in the *fp25k*-negative virus-infected cells, as most nucleocapsids were not enveloped (Fig 3A). The nucleocapsids that were not completely enveloped in ODV remained in the nucleus, rather than participated in BV formation, suggesting that the increase of BV production was not due to the nucleocapsids escaping from ODV formation.

Further investigation indicated that FP25K was related to the regulation of expression of the structural proteins, such as the major envelope protein GP64 (Fig 4A). This regulation of viral protein expression occurred at the transcriptional level (Fig 4B). We showed the up-regulated GP64 protein was incorporated into the *fp25k*-negative virions (Fig 5A). GP64 has been identified as the envelope fusion protein of group I *Alphabaculovirus* [36], which is essential for receptor recognition, cell entry, and the budding process [7, 37]. In addition, GP64 is also involved in inducing low pH-dependent membrane fusion, which is indispensable for virus entry into host cells through the endocytic pathway [38]. It was recently reported that incorporation of GP64 into the group II *Alphabaculovirus* HearNPV resulted in higher fusogenic activity and ultimately in a greater number of infectious HearNPV BVs [39], indicating that extra GP64 may benefit the infectivity of baculovirus. In our study, higher level of GP64 was detected in *fp25k*-negative BVs (Fig 5A). Other results indicate that the increase in *fp25k*-negative BV production is a consequence of higher BV infectivity, which might be the result of more GP64 being incorporated into BVs. However, we confirmed that BV infectivity was not increased when GP64 was over expressed and incorporated to BVs (Fig 6). These results suggested that the increased incorporation of BV envelope protein might not be the major cause of the enhancement of BV infectivity.

The DIP mutants lacked some genetic information, including the polyhedrin and DNA polymerase genes, and these mutations accumulated during passage in cell culture. Transposon insertion (like the FP mutants) is a crucial step in DIP mutant generation during serial passage [20, 21]. This is evidenced by a delayed production of DIP mutants during baculovirus serial passage when the transposon target sites (TTAA) were modified. These reports substantiate the idea that modification of the insertion sites contributed to the genomic stability of AcMNPV [22]. In our study, the entire sequence of the *fp25k* gene was deleted from the genome, including the TTAA sites needed for transposon insertion. The results of genomic integrity assays implied that fewer defective genomes were packaged into the *fp25k*-negative virus than the control virus (Fig 5B and 5C), suggesting that the high proportion of intact genome DNA in the *fp25k*-negative virions is likely to have led to the increase in infectivity.

**Fig 7:**
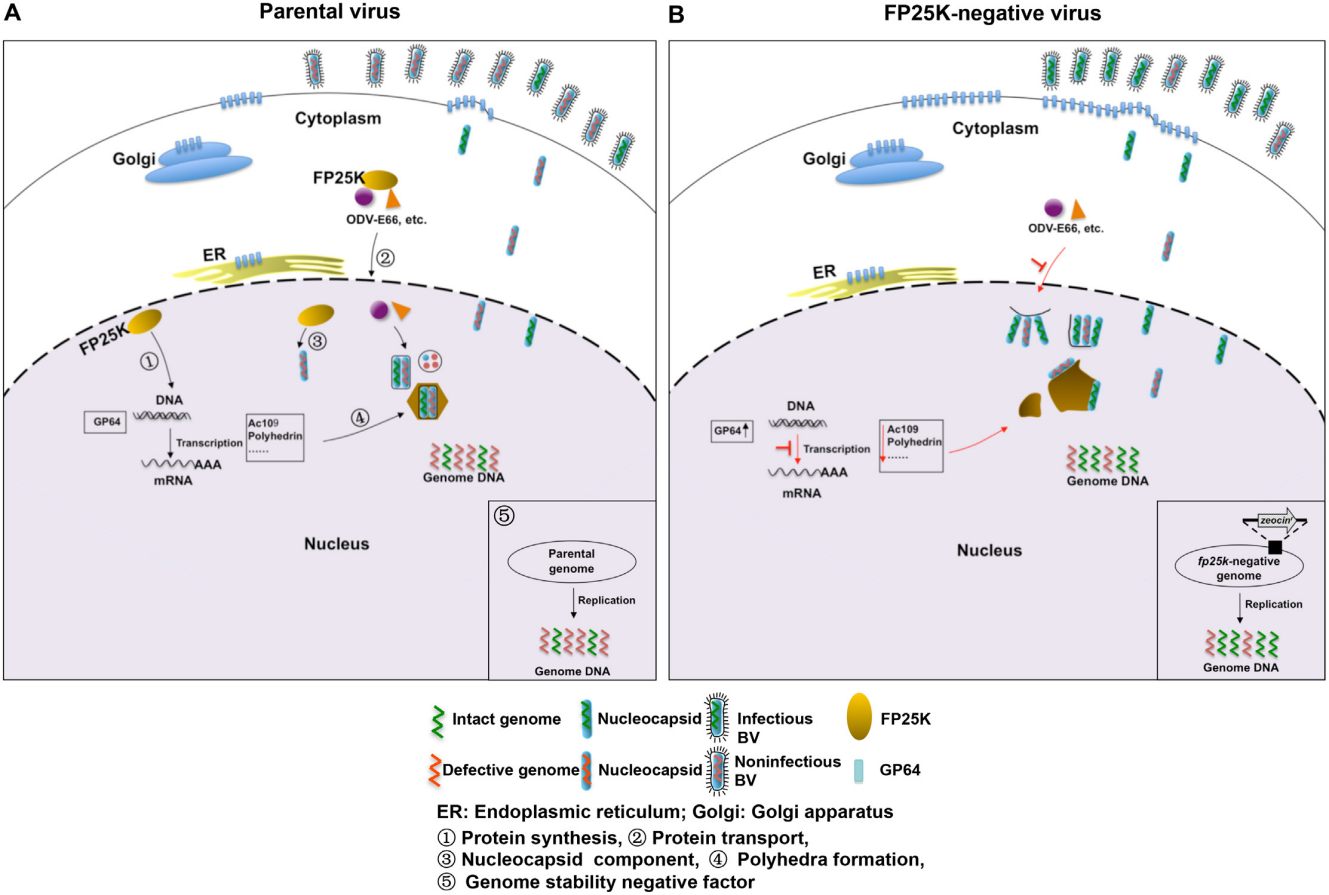
Schematic diagram of our hypothesis for parental and *fp25k*-negative AcMNPV infection. (A) In parental AcMNPV-infected cells, normal ODVs are formed in the nucleus, and most of the BVs that bud through the plasma membrane are non-infectious. (B) In *fp25k*-negative virus-infected cells, incompletely enveloped ODVs are formed, and incompletely enveloped ODVs are retained in the nucleus, while a larger number of infectious BVs are produced.

Our results suggest that the *fp25k*-negative BVs are more infectious than the parental virus, which might benefit from a higher proportion of infectious virions with better genomic integrity. Thus, we propose a model of parental and *fp25k*-negative virus infection (Fig 7). FP25K is a multifunctional protein in the life cycle of AcMNPV. FP25K participates in the protein synthesis (Fig 7A①) [17] and the transport of several structural proteins from cytoplasm to inner nuclear membrane then associated with ODV formation (Fig 7A②) [18, 19], besides being a component of the nucleocapsid (Fig 7A③) [8] and contributing the polyhedra formation (Fig 7A④) [40]. In addition, we found out that FP25K acts as a negative factor of genome stability (Fig 7A⑤), when *fp25k* was deleted a higher proportion of the newly synthesized genome DNA was intact. In AcMNPV infected cells, normal ODVs can be observed in the nucleus, and the ratio of infectious and non-infectious BVs that bud through the plasma membrane was low (Fig 7A). As for wild-type AcMNPV, the viral infectivity (copies/TCID_50_U) is about 1×10^4^, which means that 10^4^ copies of viral genome DNA result in one TCID_50_ unit [30]. In contrast, in cells infected with *fp25k*-negative virus, the expression of *gp64* is up regulated while *ac109*, and *polyhedrin* were down regulated on transcriptional level, the envelopment of nucleocapsids is incomplete. However, nucleocapsids, which are not completely occluded within ODVs, are retained in the nucleus. The deletion of *fp25k* gene results in an increase in genome stability, producing a higher proportion of infectious BVs (Fig 7B).

From an evolutionary perspective, FP mutants of baculoviruses accumulate in cell culture caused by *fp25k* mutations result in higher infectivity of BV, facilitating the transmission from cell to cell. However, the propagation of virus in insect larvae could eliminate the FP mutants, producing more OBs to benefit the spread of virus from insect to insect. A balance of BV/ODV formation will finally be achieved between FP mutants and wild type. This hypothesis could provide guidance in the application of baculovirus as different applications. FP25 mutant with higher infectivity and genome stability could be acquired through deletion of *fp25k*, which has potential to be applied as a more efficient expression vector.

In summary, our data revealed that the deletion of *fp25k* gene resulted in an increase in BV infectivity and a decrease in ODV formation. Expression of several structural proteins was regulated by FP25K at the transcriptional level. Furthermore, we demonstrated that *fp25k*-negative BVs formed with additional GP64 and greater proportion of intact genome, and that the latter one might be the major reason for the higher infectivity of *fp25k*-negative virus. These results suggest that FP25K acts as a negative factor for the infectivity of AcMNPV BVs, which give us a new insight into the FP25K-mediated regulation mechanism of BV/ODV formation, and might guide the genetic modification of baculovirus BV to be utilized as expression, surface display and gene therapy vector.
